# Cephalometric Assessment in Nonsyndromic Microtia Patients: Cross‐Sectional Study with Comparison Group

**DOI:** 10.1111/scd.70263

**Published:** 2026-09-22

**Authors:** Cléuber Roberto PEIXOTO, Bernardo OLSSON, Suyany Gabriely WEISS, Eduardo CALEME, Francielle TOPOLSKI, Rafaela SCARIOT, Alexandre MORO

**Affiliations:** ^1^ Orthodontist At Cleft Lip and Palate Integral Care Center Curitiba Paraná Brazil; ^2^ Oral and Maxillofacial Surgeon At Cleft Lip and Palate Integral Care Center Curitiba Paraná Brazil; ^3^ Department of Stomatology Federal University of Paraná Curitiba Paraná Brazil; ^4^ Periodontist At Cleft Lip and Palate Integral Care Center Curitiba Paraná Brazil; ^5^ School of Health Sciences, Postgraduate Program in Dentistry Universidade Positivo Curitiba Paraná Brazil; ^6^ Department of Orthodontics Federal University of Paraná Curitiba Paraná Brazil

**Keywords:** Hemifacial microsomia, Microtia, Orthodontics, Cephalometry

## Abstract

**Aims:**

To assess and compare the cephalometric characteristics of patients with nonsyndromic microtia.

**Methods and Results:**

This cross‐sectional comparative study evaluated the cephalometric characteristics of 16 patients with nonsyndromic microtia and 48 patients without microtia (n = 48). A retrospective analysis was performed using lateral cephalograms, orthopantomograms, dental casts, facial photographs, and intraoral photographs. ANB (*p* = 0.044), FMA (*p *< 0.001), SN.GoGn (*p* = 0.003), Y‐axis (*p* = 0.024), LAFH (*p *< 0.001), and 1‐NB (*p* = 0.014) were higher in the microtia group, whereas 1.NA (*p* = 0.018) and IMPA (*p* = 0.029) were lower relative to controls. Twelve patients had deviation of the mandibular midline to the microtia side; one patient had left‐sided microtia and deviation of the midline to the right side, and three patients did not exhibit mandibular midline deviation.

**Conclusion:**

Patients with microtia showed prevalence of a Class II skeletal and dolichocephalic pattern, predisposition to upper incisor movement in the vertical direction, and lower incisor protrusion. In addition, they showed a tendency toward deviation of the lower midline to the affected side.

## 1 Introduction

Microtia is a congenital deformity characterized by external auditory canal (EAC) underdevelopment, with severity ranging from mild structural abnormalities to complete absence of the ear [1, 2]. Microtia is classified into 4 types [3]: Grade 1 is the mildest type in which the affected ear is slightly smaller but maintains all its structures, with an underdeveloped ear canal but no hearing loss; Grade 2 is characterized by partial ear development, with a narrow ear canal and possible hearing loss; Grade 3 is characterized by possible absence of the EAC and of the tympanic membrane, with the external ear identified only by a peanut‐shaped lobe, and total absence of the ear canal, known as aural atresia; and Grade 4, the most severe type, is characterized by total absence of the EAC, a rare type of atresia known as anotia [4].

Microtia can occur as an isolated birth defect, part of an anomaly, or a syndrome, such as Goldenhar, Treacher Collins, Nager, and Townes‐Brocks syndromes [5, 6]. Its etiology remains poorly understood, but some evidence implicates genetic and environmental factors [6, 7]. Certain risk factors for microtia are eclampsia or preeclampsia, anemia, ethnicity, advanced maternal or paternal age, multiple pregnancies, mother with type 1 diabetes mellitus, influenza during the first trimester of pregnancy, use of medications such as isotretinoin or mycophenolate mofetil, and hypervitaminosis A [8, 9, 10, 11].

Evidence suggests that genetic factors predominate in embryonic craniofacial morphogenesis, whereas environment influences primarily determine postnatal facial morphology, especially during facial growth [12, 13]. The main hypotheses underpinning microtia development are abnormal neural crest cell mitigation and vascular disruption (hematoma of the stapedial artery) [14]. Evidence concerning craniofacial growth suggests patients with microsomia have abnormal craniofacial growth anteroposteriorly and transversely [15]. Craniofacial microsomia types are characterized by active and variable growth on the dysplastic side, following a distinct pattern from other mandibular growth disorders [16].

According to data from the Latin American Collaborative Study of Congenital Malformations (ECLAMC), the microtia prevalence rate is 1 per every 5000 births and is more common among the Eastern and Hispanic population. However, no prevalence data are available for Brazil. Treatment options for microtia include ear reconstruction via reparative surgery using the costal cartilage of the eighth rib [17] or placement of percutaneous titanium implants, with silicone prostheses designed in 3D planning software and manufactured using a 3D printer [18].

Regarding the development and measurement of facial bones, mainly the maxilla and mandible, studies considering patients with microtia remain unavailable. Therefore, this study aimed to evaluate cephalometric measurements in patients with nonsyndromic microtia. We also tested the hypothesis that the mandibular dental midline deviates toward the microtia‐affected side.

## 2 Materials and Methods

### 2.1 Ethical Considerations

This study was approved by the local committee 31159520.6.0000.0093 and followed the Helsinki Declaration. All patients with microtia signed an informed consent form.

### 2.2 Study Design and Participants

This cross‐sectional comparative study included patients selected from 2 databases. Patients with nonsyndromic microtia (microtia group) were selected by convenience/consecutive sampling from Complexo Hospitalar do Trabalhador/Centro de Atendimento Integral ao Fissurado Lábio Palatal (CHT/CAIF, Curitiba, PR) database, whereas patients without microtia (comparison group) were selected from the American Association of Orthodontists database. The following inclusion criteria were applied for patients with microtia: patients of any age, both sexes, nonsyndromic, with microtia who had lateral—both sides—and frontal facial photography, digital lateral teleradiographs, who needed orthodontic treatment, and patients who have not undergone previous facial orthopedic/orthodontic treatment. The exclusion criteria were syndromic patients, patients with orofacial clefts (cleft lip and/or palate), and previous orthognathic surgery.

The comparison group was derived from the American Association of Orthodontists database using a 1:3 matching ratio (1 patient with microtia, 3 patients without microtia) and was matched for sex, age, and type of Angle's skeletal malocclusion. The 1:3 ratio was chosen to increase the statistical power and precision of estimates. The exclusion criteria were the same for both groups.

### 2.3 Data Collection

This single‐phase study, conducted from April 2019 to December 2020, included lateral cephalograms, orthopantomograms, dental casts, medical records, facial photographs, and intraoral photographs.

Clinical data included sex, age (in complete years), microtia‐affected side, and midline deviation. Microtia severity was graded from 1 to 4 following Hunter et al. (2009).

For cephalometric analysis, lateral cephalometric radiographs in the microtia group were taken at 2 dental radiology centers using an Orthopos XG 5 orthopantomograph (Sirona Dental Systems) under standard conditions (84 kVp, 13 mA, exposure time 9.4 s, 1.52 m focus sensor distance, and 1:1 magnification factor) for all images.

Lateral cephalometric radiographs of patients in the microtia and comparison groups were saved directly in the CEF X program (CDT, Cuiabá, Brazil), version 4.5.10, using 244 dpi and a constant magnification of 10%. The angular and linear measurements (Table 1) were digitally obtained by a single trained examiner.

**TABLE 1:** Description of angular cephalometric measurements and linear cephalometric measurements obtained.

	NAME	DEFINITION
Angular	SNA	Angle formed by the SN line to the NA line, indicates the position of the maxilla in the posterior‐anterior direction to the skull base
	SNB	Angle formed by the SN line to the NB line, indicates the position of the mandible in the posterior‐anterior direction to the base of the skull
	ANB	Angle formed by the line NA and NB, indicates the maxilla‐mandible relationship in the anteroposterior
	FMA	Angle formed by the Frankfurt and mandibular planes, indicates vertical growth pattern
	SN.GoGn	Angle formed by lines SN to GoGn, indicates vertical growth pattern
	Axis Y	Angle formed by the SN to Gn line, indicates vertical growth pattern
	SN.Oc	Angle formed by the SN line and the occlusal plane, indicates the vertical position of the occlusal plane in relation to the anterior base of the skull
	1.SN	Angle formed by the SN line with the long axis of the upper incisor in relation to the skull base
	1.NA	Angle formed by the NA line with the long axis of the upper incisor, indicates the axial inclination of this tooth with its bone base
	1.NB	Angle formed by the line NB with the long axis of the mandibular incisor, indicates the axial inclination of the mandibular incisor with its bone base
	IMPA	Angle formed by the long axis of the lower incisor with the mandibular plane, indicates the inclination of the mandibular incisor in relation to its bone base
	Interincisive Angle	Angle formed by the intersection of the long axes of the upper and mandibular incisors, indicates the degree of protrusion of the incisors
	ANL	Angle formed by the base of the nose with the upper lip, indicates the position of the maxilla at the base of the skull.
Linear	WITS	Sagittal jaw relationship assessed by projecting points A and B onto the occlusal plane (AO–BO distance).
	LAFH	Lower Anterior Facial Height (ANS‐Me)
	1‐NA	Linear distance between the upper incisor to the NA line
	1‐NB	Linear distance between mandibular incisor to line NB
	Upper lip—Line E	Linear distance between the upper lip to Line E
	Lower lip‐Line E	Linear distance between the lower lip to Line E

**TABLE 2:** Median age (minimum and maximum) age according to sex.

		n (%)	Med. age (min–max)	*p* ^*^
Sex	Male	44 (69)	14 (7–35)	0.484
	Female	20 (31)	13 (10–17)	

Abbreviations: Med = median; Min = minimum; max = maximum.

* Mann‐Whitney U test.

### 2.4 Statistical Analysis

The data were subjected to descriptive and inferential analyses using the Statistical Package for Social Science program (SPSS, version 27.0; SPSS Inc., Chicago, IL, USA), with a 95% confidence interval. The cephalometric measurement values were subjected to the Shapiro–Wilk test for normality, and nonparametric variables were described as median, minimum, and maximum. The Mann–Whitney U test was used to compare the median age between the microtia and comparison groups. Additionally, it was applied to evaluate differences in cephalometric variables between the two groups.

## 3 Results

**FIGURE 1:**
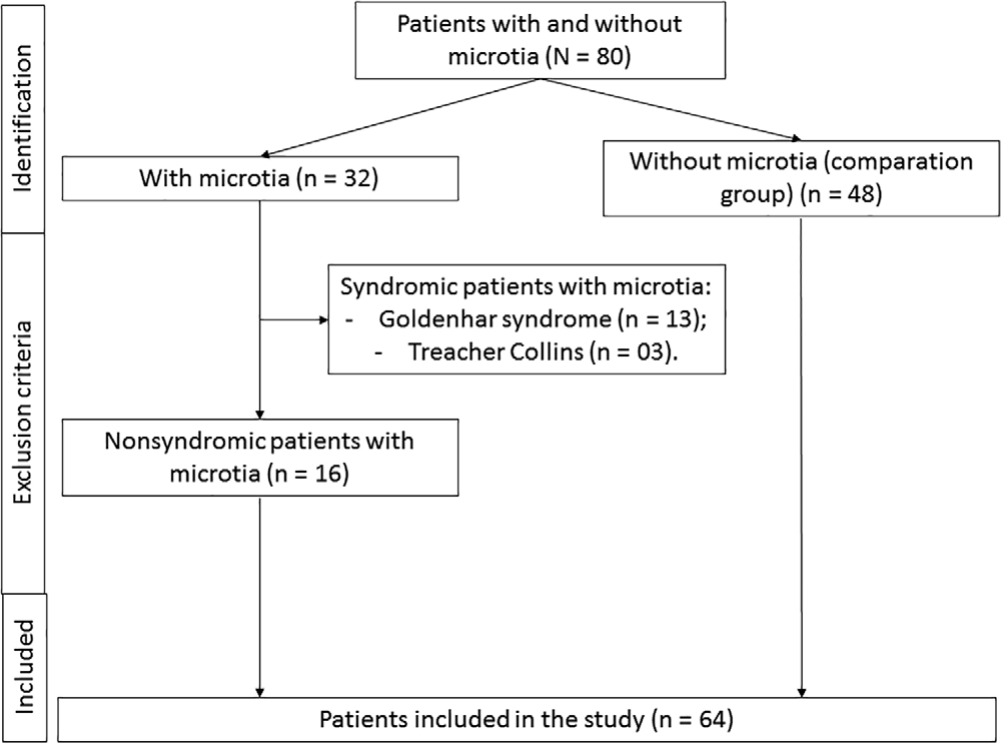
Flowchart showing the final sample composition.

In total, 64 patients were included in this study: 16 in the microtia group and 48 in the comparison group (Figure 1).

The microtia group comprised 11 males and 5 females. The median (minimum–maximum) age was 21 years (7–35). The Mann–Whitney U test demonstrated no statistically significant difference in age between males and females (*p* = 0484) (Table 2).

Regarding dental malocclusion, 7 patients had Angle's Class I malocclusion, and 9 patients had Class II malocclusion on the microtia‐affected side. Microtia was classified as Grade 1 in 1 patient, Grade 3 in 13 patients, and Grade 4 in 2 patients.

The comparison group comprised 48 patients (33 males and 15 females), with a median (min–max) age of 21 years (7–35), and 21 patients were classified as dental malocclusion Class I and 27 patients as Class II.

The cephalometric analysis revealed that the microtia group exhibited higher values of ANB (*p* = 0.044), FMA (*p *< 0.001), SN.GoGn (*p* = 0.003), Y‐axis (*p* = 0.024), and LAFH (*p *< 0.001), and lower values of 1‐NB (*p* = 0.014), 1.NA (*p* = 0.018), and IMPA (*p* = 0.029) relative to the control. No significant differences existed in the other cephalometric measurements. Table 3 lists all the result data.

**TABLE 3:** Median (minimum and maximum) cephalometric measurements in patients with microtia and comparison group (adapted from Steiner, Tweed, Downs, Wits, and Ricketts Analyses).

Measure	Microtia group	Comparison group	*p* value^*^
Angular	Median (min–max)	Median (min–max)	
SNA	81.33 (75.94–87.77)	79.94 (75.72–91.84)	0.402
SNB	77.04 (69.99–85.49)	78.61 (68.54–87.16)	0.474
ANB	4.44 (−1.06–10.06)	3.62 (−0.55–9.58)	**0.044**
FMA	29.05 (18.41–41.95)	21.06 (7.69–32.39)	**<0.001**
SN.GoGn	37.18 (29.09–47.57)	31.72 (14.48–42.22)	**0.003**
Y axis	69.54 (62.30–77.59)	67.08 (59.14–73.18)	**0.024**
SN.Oc	19.13 (9.03–40.91)	17.71 (−3.56–26.16)	0.420
1.SN	99.23 (84.82–116.97)	102.44 (92.67–114.72)	0.063
1.NA	17.51 (2.96–33.48)	20.84 (6.93–31.29)	**0.018**
1.NB	22.23 (13.80–29.73)	23.33 (5.12–33.56)	0.921
IMPA	84.42 (79.10–94.61)	90.43 (74.90–101.93)	**0.029**
Interincisive Angle	133.76 (124.75–152.66)	130.94 (115.36–159.97)	0.361
ANL	109.51 (86.43–128.98)	107.91 (76.89–142.84)	0.976
Linear			
WITS	1.48 (0.01–9.87)	1.99 (0.02–9.5)	0.951
LAFH	71.39 (50.48–97.46)	59.04 (45.51–78.76)	**<0.001**
1‐NA	2.85 (−1.96–8.92)	3.13 (−0.80–7.20)	0.626
1‐NB	4.97 (0.25–12.66)	3.94 (−0.33–7.67)	**0.014**
Upper lip‐Line E	−2.25 (−6.5–2.0)	−2.0 (−5.00–4.5)	0.909
Lower lip‐Line E	−0.50 (−6.0–4.5)	−0.50 (−9.50–4.5)	0.743

Abbreviations: Med = median; Min = minimum; max = maximum.

* Mann‐Whitney U test with a significance level of 0.05.

Regarding the side of mandibular midline deviation and microtia‐affected side, 7 and 5 patients presented with both microtia and mandibular midline deviation on the right and left sides, respectively; one patient had left‐sided microtia and midline deviation to the right side, and 3 patients showed no mandibular midline deviation.

## 4 DISCUSSION

Microtia grade and middle ear abnormality severity are correlated, and better development likely reflects well‐developed middle ear structures [1, 10, 19]. The existence of additional structural anomalies suggests a broader developmental problem in most patients with microtia [19, 20]. Therefore, from a clinical perspective, conducting a thorough and detailed evaluation in this regard is necessary. This should include careful assessment of facial morphology, given the proximity and/or common origin of the embryonic tissue that gives rise to the external and middle ear structures, maxilla, and mandible.

To date, the cephalometric characteristics of patients with microtia remain unexamined. Our findings showed that patients with microtia presented a tendency toward a Class II skeletal pattern with vertical growth and posterior mandible inclination, movement of upper incisors in the vertical direction, and protrusion and buccal inclination of the mandibular incisors.

Approximately 20%–60% of patients with microtia have associated anomalies or an identifiable syndrome [21]. Therefore, patients with microtia should be screened for other dysmorphic physical characteristics [20]. Although none of the patients had syndromes, mandibular growth alterations were likely present, resulting in a more hyperdivergent facial phenotype. The alteration degree was likely small since none of the patients had significant facial asymmetry. Nevertheless, this alteration could be associated with lower midline deviation because 12 patients in our sample presented with both microtia and mandibular midline deviation to the same side.

Patients with syndromic microtia were intentionally excluded from this study to maintain homogeneity and enhance the specificity of our findings. Syndromic microtia (e.g., associated with oculoauriculovertebral spectrum/Goldenhar syndrome, Treacher Collins syndrome, Nager syndrome, Townes‐Brocks syndrome, or other genetic conditions) frequently involves distinct etiological mechanisms, multisystem anomalies, and additional craniofacial or extracranial features that differ substantially from those observed in isolated (nonsyndromic) microtia [22]. Including syndromic cases could introduce significant confounding and heterogeneity, potentially masking associations or patterns specific to nonsyndromic microtia. This focused approach is consistent with many previous studies in the field that restrict enrollment to nonsyndromic cases to better characterize risk factors, clinical features, and outcomes unique to isolated microtia. While stratified analyses including syndromic patients would be valuable in larger multicenter investigations, they were beyond the scope and primary objectives of the present study.

After evaluating the relationships between mandibular morphology and temporal bone anomalies using CT images, Takano et al. (2016) showed that mandibular development was significantly correlated with aeration of the middle ear space, pneumatization of mastoid air cells, and formation of the oval window, but not with the presence of a bony part of the EAC or with ossicular development [20]. They also found that ear anomalies may worsen as mandibular dysplasia becomes more severe. In this study, microtia was classified as Grade 3 in 13 patients and Grade 4 in two patients, indicating major ear anomalies; however, mandibular growth alteration was less pronounced and did not appear proportional to ear defect severity. These observations indicate that mandibular growth regulation is multifactorial and remains difficult to fully elucidate.

The mandibular growth changes in patients with microtia may explain the observed alterations. All patients showed a visible change in the condyle, which is located close to the ear and is considered a key mandibular growth center [15]. Alterations in temporomandibular joint morphology may affect craniofacial complex development potentially resulting in accelerated maturation, especially in areas of cartilage compression. The growth of mandibular condylar cartilage is more influenced by extrinsic than intrinsic factors, unlike the epiphyseal cartilage in long bones, [23, 24] consolidating the tendency toward deviation of the lower midline to the same side affected by microtia. These findings suggest possible alterations in transverse mandibular growth, consistent with previous reports [25].

Dental alterations in the microtia group represent dentoalveolar compensations for alterations in maxillary and mandibular growth. These alterations have already been described in the orthodontic literature for Class II hyperdivergent patients [26]. The cephalometric evaluation of patients with nonsyndromic microtia reveals craniofacial morphological alterations, such as mandibular asymmetries, occlusal plane canting, or mild maxillomandibular discrepancies. These findings hold significant clinical importance, as they support more precise multidisciplinary treatment planning, orthodontic management, and long‐term monitoring for potential occlusal disturbances, masticatory dysfunction, or airway implications. By characterizing the specific cephalometric pattern associated with isolated microtia, this study helps differentiate these patients from those with no microtia, thereby facilitating earlier, more tailored interventions and providing valuable reference data for future research on craniofacial growth trajectories and aesthetic/functional outcomes.

Concerning sex, 11 patients were male (5 with microtia on the right side and 6 with micro on the right side and 2 with microtia on the left side). This establishes a clear difference between the present research and published studies, states that microtia occurs more frequently in males and on the right side [26]. Despite the small sample size, these findings suggest possible variations among microtia patients.

This study, however, has some limitations. The sample size was relatively small (*n* = 16 patients with microtia), which is common in research on rare craniofacial anomalies such as nonsyndromic microtia due to its low prevalence and the stringent inclusion criteria required for reliable cephalometric analysis (e.g., specific imaging modalities and absence of prior interventions). This modest sample may reduce statistical power to detect smaller effect sizes, increase the risk of type II errors, and limit the findings’ generalizability to broader populations. Nevertheless, the use of a matched 1:3 comparison group from a large database helped mitigate some power concerns, and the observed differences in cephalometric variables were consistent with patterns reported in the prior literature on microtia and related craniofacial conditions. Future multicenter or registry‐based studies with larger cohorts would be valuable for confirming and extending these results. Ideally, 3D cephalometric images should be used to assess whether midline deviation is associated with asymmetric growth of the maxilla and mandible. This analysis would allow assessment of whether any morphological changes exist in mandibular condyles. Furthermore, due to the small sample size (*n* = 16) and the uneven distribution of microtia grades in our nonsyndromic cohort—consistent with the predominance of more severe forms (Grades 3–4) and the relative rarity of Grade 1 in isolated cases—no formal subgroup analysis by microtia severity was performed. Future larger‐scale studies are warranted to explore potential grade‐specific variations.

Finally, microtia should be further investigated to improve the current approach to functional and aesthetic corrections, appreciating psychosocial support and multidisciplinary team participation (otolaryngologists, dentists, ophthalmologists, speech therapists, and psychologists), greatly facilitating the diagnosis and early intervention of orthodontics and dentofacial orthopedics, contributing to enhanced treatment and prognosis, minimizing social stigma, and allowing patients and their families to improve quality of life.

In conclusion, patients with microtia evaluated in this study have a Class II and hyperdivergent skeletal pattern, with a predisposition to upper incisor movement in the vertical direction and protrusion and inclination of mandibular incisors.

## Funding

The authors have nothing to report.

## Ethics Statement

This study was approved by the local committee (31159520.6.0000.0093) and followed the Helsinki Declaration. All patients with microtia signed an informed consent form.

## Conflicts of Interest

The authors declare no conflicts of interest.
